# Intron Retention: A Reemerging Paradigm in RNA Biology and Post-Transcriptional Gene Regulation

**DOI:** 10.3390/genes16080986

**Published:** 2025-08-21

**Authors:** Ana L. Porras-Tobias, Abigail Caldera, Isabel Castro-Piedras

**Affiliations:** Center for Biotechnology and Genomics, Texas Tech University, Lubbock, TX 79409, USA; aporrast@ttu.edu (A.L.P.-T.); abigcald@ttu.edu (A.C.)

**Keywords:** intron retention, IR, IR dysregulation, alternative splicing, AS, nonsense-mediated decay, NMD, cancer, neurodegeneration, aging

## Abstract

For 40 years, Intron Retention (IR) was dismissed as splicing noise and is now recognized as a dynamic and evolutionarily conserved mechanism of post-transcriptional gene regulation. Unlike canonical splicing, which excises all introns from pre-mRNAs, IR selectively retains intronic sequences, albeit at seemingly random places; however, current research now reveals that this process is strategic in its retention. IR influences mRNA stability, localization, and translational potential. Retained introns can lead to nonsense-mediated decay, promote nuclear retention, or give rise to novel protein isoforms that contribute to expanding proteomic and transcriptomic profiles. IR is finely regulated by splice site strength, splicing regulatory elements, chromatin structure, methylation patterns, RNA polymerase II elongation rates, and the availability of co-transcriptional splicing factors. IR plays critical roles in cell-type and tissue-specific gene expression with observed patterns, particularly during neuronal, cardiac, hematopoietic, and immune development. It also functions as a molecular switch during cellular responses to environmental and physiological stressors such as hypoxia, heat shock, and infection. Dysregulated IR is increasingly associated with cancer, neurodegeneration, aging, and immune dysfunction, where it may alter protein function, suppress tumor suppressor genes, or generate immunogenic neoepitopes. Experimental and computational tools like RNA-seq, RT-PCR, IRFinder, and IntEREst have enabled transcriptome-wide detection and validation of IR events, uncovering their widespread functional roles. This review will examine current knowledge on the function, regulation, and detection of IR, and also summarize recent advances in understanding its role in both normal and pathophysiological settings.

## 1. Introduction

Intron retention (IR) is a form of Alternative Splicing (AS) where introns are deliberately retained in mature mRNAs, contrasting with conventional splicing that removes all introns prior to export and translation [1,2,3]. IR is a significant mechanism of post-transcriptional gene regulation, influencing processes like stress response, cell cycle control, differentiation, and apoptosis. Additionally, it contributes to transcriptome and proteome diversity, either by generating novel protein isoforms or regulating mRNA stability through decay or nuclear and cytoplasmic retention [1,2,3]. IR is especially important in species with fewer protein-coding genes, where it supplements functional AS. It also plays a pivotal role in disease, with aberrant IR patterns linked to cancer progression, immune dysregulation, and aging [1,2,3]. Altogether, IR is now recognized as a widespread and dynamic regulatory tool with far-reaching implications in both health and disease.

Unfortunately, the question of why some introns are retained and why others are not is still unknown [4]. Moreover, the patterns of cis- and trans-acting components are still being uncovered [2,5,6,7,8,9,10], and the interactions between spliceosome subunits and transcripts remain a puzzle still to be solved. There is still critical work to be done to illuminate the world on IR patterns and processes.

This review presents current findings on the function, regulation, detection, and consequences of IR, highlighting crucial research breakthroughs in development, stress adaptation, experimental validation, and disease pathogenesis. Additionally, it traces the progression of the recognition of IR from a presumed splicing error to a strategic cellular post-transcriptional mechanism. Using the evolving technological advances, IR can be studied at a greater capacity than before [11,12]. Understanding IR not only expands our view of AS but also provides new insights into transcriptome regulation and potential therapeutic targets.

## 2. IR Regulation: A Complex, Multifactorial Process

Before discussing IR, it is important to revisit the mechanism of canonical RNA splicing. In most eukaryotic cells, RNA splicing is a fundamental post-transcriptional mechanism that removes non-coding introns from precursor messenger RNA (pre-mRNA) and joins together protein-coding exons to produce a mature, translatable mRNA transcript. This process is essential for accurate gene expression and proteome integrity [13].

**Figure 1:**
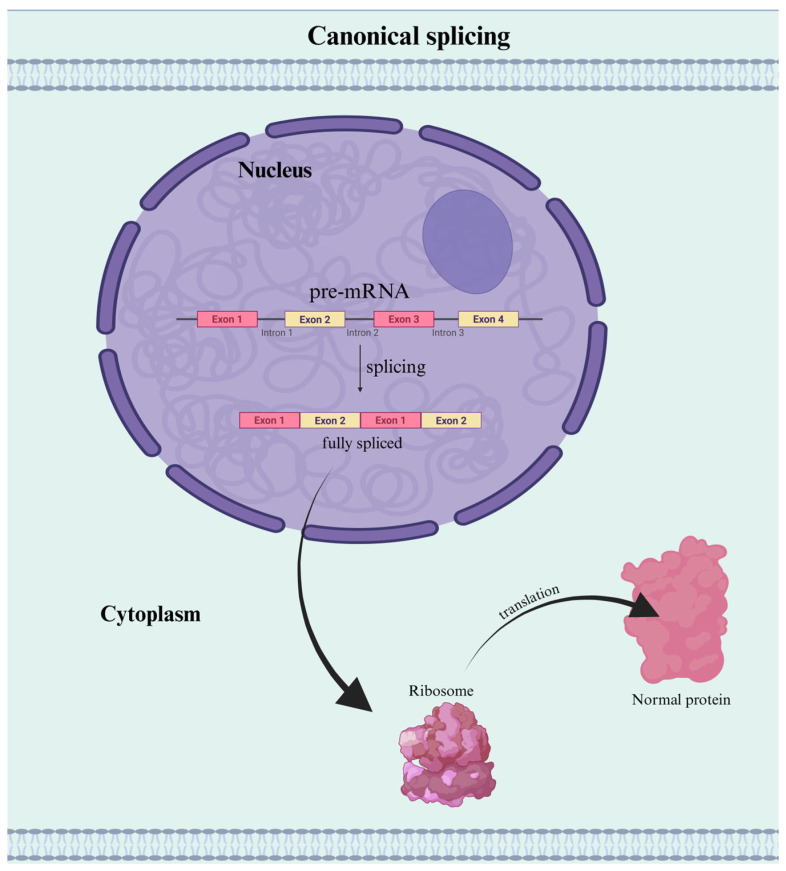
Normal Pre-mRNA Splicing and Translation. In canonical mRNA processing, introns are accurately removed from the pre-mRNA transcript by the spliceosome, and exons are joined to form a continuous coding sequence. The fully spliced mRNA is exported to the cytoplasm, where it is translated by ribosomes into a full-length, functional protein. Created in BioRender. Ana L. Porras-Tobias. (2025) https://app.biorender.com/illustrations/6875ddce5c9667b402ea742e.

Splicing is catalyzed by the spliceosome, a dynamic ribonucleoprotein (RNP) complex composed of five small nuclear RNAs (snRNAs) and numerous associated proteins. This molecular machine recognizes conserved splice sites at the 5’ and 3’ ends of introns, as well as a branch point sequence, to carry out two sequential reactions: (1) cleavage at the 5’ splice site and formation of a lariat structure at the branch point, and (2) cleavage at the 3’ splice site followed by ligation of adjacent exons to form a continuous coding sequence (Figure 1) [13].

While canonical splicing typically results in complete intron removal, AS introduces variability by allowing pre-mRNA to be spliced in multiple ways. This process enables the production of diverse mRNA isoforms from a single gene. Among the various types of AS, IR is a distinct and recognized mechanism [13].

IR regulation follows a recognizable pattern. Retained introns are often shorter, GC-rich, and flanked by weak splice sites, all of which make them less likely to be removed during splicing [14,15]. Overall, the regulation of IR is presented as a multifactorial process, integrating cis-elements, trans-factors, and epigenetic marks to fine-tune gene expression [3]. These findings suggest that the sequence plays a key role in the regulation of IR, including splice site strength, sequence motifs, and Splicing Regulatory Elements (SRE). Moreover, these features are influenced by epigenetic mechanisms, including chromatin structure, DNA methylation, and RNA polymerase II elongation rate [3,16,17].

### 2.1. Sequence-Dependent IR Regulation

**Figure 2:**
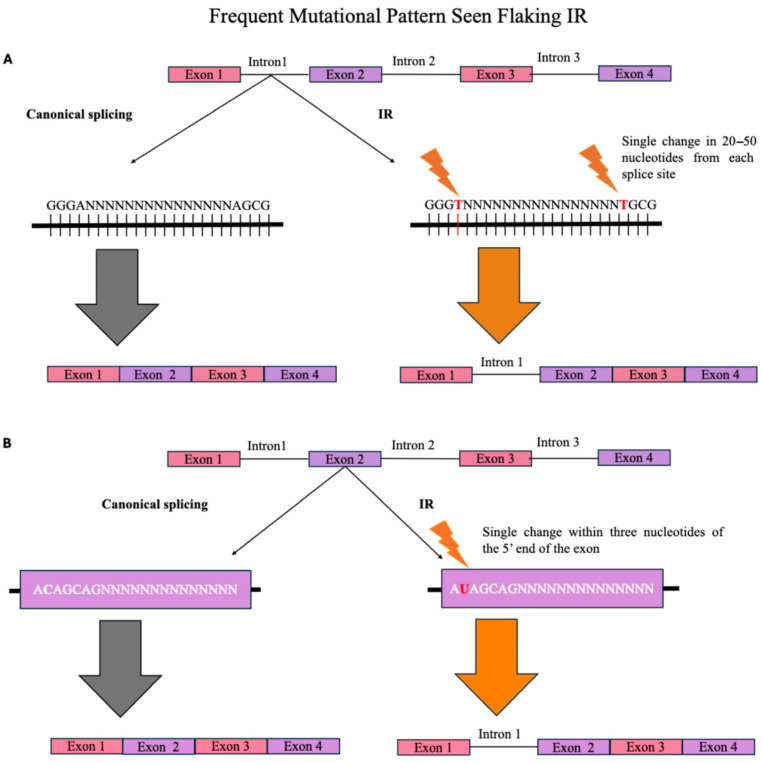
Frequent Mutational Pattern Seen Flanking IR. This figure depicts the canonical pattern found in IR mRNA across different species. (**A**). The retained intron is characterized by a high GC content; a single change in each splice site (5’ and 3’) within 20–50 nucleotides from the splice site usually involves an A to T transition (weak 5’ and 3’ splice site within the intron). This often creates a premature stop codon, triggering a signal to retain the intron. (**B**). In the flanking exon, a mutation within the first three nucleotides of the 5’ splice site results in intron retention (weak 5’ splice site within the exon). In some instances, the presence of weak 5’ and 3’ splice sites of the intron will result in IR, and with others, IR further requires the mutations in the 5’ exon to produce IR products. The cause of the mutations is still unknown and needs further experimentation. Mutations are denoted with an orange lighting bolt.

Sequence-dependent IR regulation may be divided into several categories: (a) Splice Site Strength, (b) Presence of Transcription Factor Motifs, and (c) Presence of Splicing Regulatory Elements. Splice site strength is considered a key regulatory feature driving IR in tandem with other regulatory elements. IR is strongly associated with weak 5’ and 3’ splice sites, which hinder proper intron excision (Figure 2) [8,18,19,20,21,22,23]. A single nucleotide change within 20–50 nucleotides in the intron from both 5’ and 3’ splice sites can generate a Premature Termination Codon (PTC), which signals a binding protein to retain the intron [8,23]. In some cases, the neighboring exon must include a mutation within three nucleotides of the 5’ splice site for the intron to be retained [24]. One example is in spermatogenesis or the immune response, which often displays suboptimal splice site sequences, thereby reducing splicing efficiency [20]. Mutations in one of the sites still result in full intron removal, confirming the need for both splice site mutations to allow for IR products [23].

Another significant sequence-dependent regulation to consider is the presence of intragenic and promoter-associated motifs, which influence IR dynamics in a context-dependent manner. Transcription Factors (TFs) are potential splicing enhancers with sequence motifs assisting in regulating IR through binding sites [25]. Ullah et al., identified a significant enrichment of C2H2 zinc finger TF motifs in introns with elevated IR readings, which suggests these motifs may function as splicing enhancers [25]. Using RNA-seq datasets from the developmental stages of saffron stigma, Ahrazem et al., concluded that IR is an important form of AS [5]. In the saffron *CsCCD2* gene, IR was predominant in developmental stages and tissues lacking crocetin accumulation, correlating with specific cis-regulatory promoter motifs responsive to light, temperature, and circadian cues [5].

Additionally, another well-studied component of IR regulation is the role of splicing regulatory elements [7,8,23,24,25,26,27,28,29,30,31,32]. It identifies exonic and intronic enhancers and silencers, such as purine-rich exonic splicing enhancers and the GGG triplet, as key regulators of IR by promoting or repressing splice site recognition [8,23]. Several RNA-binding proteins, including hnRNPLL, PTBP1, and ASF/SF2, are shown to bind specific SREs, altering IR patterns in a cell-specific manner, allowing stimulation of splicing processes (Table 1) [7,24,27,28,30,31,32]. Therefore, these RNA-binding proteins (RBPs) play a key role in IR regulation through direct interactions with pre-mRNAs. Proteins like PTBP1, SFPQ, hnRNPLL, Chtop, PABPN1, and Nova-1 modulate IR by promoting or inhibiting spliceosome access, influencing transcript stability, localization, or degradation [27,28,31,33,34].

Polypyrimidine tract-binding protein (PTBP1) is a key component in the complex regulation of IR that leads to different fates for intron-retaining transcripts, such as nuclear sequestration, decay, or translation, depending on the cell context [30,35]. PTBP1 regulates IR by controlling the splicing of 3’ terminal introns in nonneuronal cells, preventing the export of incompletely spliced mRNAs [30]. PTBP1 represses the export of these mRNAs to the cytoplasm, triggering their nuclear degradation through the nuclear RNA surveillance machinery, including the translocated promoter region protein and the exosome complex [30]. During neuronal differentiation, reduced PTBP1 expression enables proper splicing of regulated introns, allowing the accumulation of functional mRNAs in the cytoplasm, which is crucial for the activation of neuron-specific genes [30]. A specific example of PTBP1’s key role in promoting IR is in the silencing of the fosB intron 4 splicing [31]. PTBP1’s influence on IR leads to the production of a truncated DFosB protein, contributing to its accumulation in response to chronic stimuli [31]. This highlights PTBP1’s regulatory role in controlling the *FosB* gene expression by modulating IR, which is a crucial step in the formation of DFosB in response to stressors [31].

Splicing Factor Proline and Glutamine-rich (SFPQ) mutations are linked to the pathogenesis of Amyotrophic Lateral Sclerosis (ALS) [33]. These mutations highlight the role of the predominantly expressed RNA processing regulators in motor neurons [33]. The most notable IR occurs in the SFPQ transcript, where the SFPQ protein binds to the retained intron, shows reduced nuclear presence in valosin-containing protein mutant cultures, and is lost from the nuclei of motor neurons in both mouse models and human sporadic ALS [33]. These findings establish SFPQ IR and nuclear loss as molecular attributes of both familial and sporadic ALS [33].

Heterogeneous nuclear ribonucleoprotein (hnRNPLL) governs lineage-specific IR during T cell development [28]. By examining RNA from T cells with an inactivating *Hnrpll* gene mutation and B lymphocytes that downregulate *Hnrpll* during differentiation, Cho et al. demonstrated that hnRNPLL induces retention of introns flanking exons 4 to 6 in *Ptprc* mRNA encoding the tyrosine phosphatase CD45 [28]. Similar patterns of hnRNPLL-induced differential IR were identified in 14 other genes, providing new insights into the hnRNPLL splicing program in T cells [28].

Chromatin Target of Prmt1 (Chtop) is a regulator of transcription and mRNA export controlled through an autoregulatory negative feedback loop, where it binds its mRNA to retain intron 2 during splicing [34]. This results in a PTC, leading to nonsense mediated Decay (NMD) of the mRNA [34]. NMD is a quality control mechanism that functions as a checkpoint for gene expression where it protects cells from the consequences of gene mutations and errors during translation. Specifically, NMD selectively degrades RNAs containing PTCs that prematurely terminate translation, such as nonsense, frameshifts, and some splice-site mutations [36,37]. Chtop interacts with exon 2 and intron 2 of its mRNA via specific domains, and the splicing of intron 2 is modulated by Chtop and hnRNP H in an antagonistic manner, providing a novel mechanism for the regulation of mRNA and protein levels via IR [34].

Mutations in the *PABPN1* gene, which encodes poly(A)-binding protein nuclear 1 (PABPN1), are shown to cause oculopharyngeal muscular dystrophy, a late-onset disorder with an unclear molecular basis [27]. PABPN1 autoregulation relies on inefficient splicing of its 3’-terminal intron, where PABPN1 binds to an adenosine-rich region in its 3’-untranslated region (UTR) to promote IR [27]. This process is coupled with nuclear pre-mRNA decay via the exosome, highlighting a regulated IR mechanism that controls PABPN1 expression homeostasis [27].

Alternative Splicing Factor or Splicing Factor-2 (ASF/SF2) is crucial in the synthesis of endoglin in endothelial cells [7]. An imbalance of S-endoglin and L-endoglin isoforms can cause senescence, which contributes to the aging of the cell [7]. ASF/SF2 was shown to favor the synthesis of the S-endoglin isoform in the aging endothelial cell [7]. It was demonstrated that when the cell reaches the senescence stage, ASF/SF2 translocates to the cytoplasm to intercept the minor spliceosome and causes the retention of intron 14 [7]. This upregulates S-endoglin mRNA and leads to the production of the senescent isoform [7]. This example highlights how AS through IR functions as a key regulatory mechanism in gene expression, especially during cellular aging.

Additionally, the regulation by splicing factors plays a key role in regulating IR, impacting gene expression during developmental and stress conditions. The splicing factor U2AF1 was shown to induce IR in the gene *CPNE1*, promoting cellular senescence, indicating a direct functional consequence of splicing factor activity and other regulators like UAF-1 and SFA-1, which influence IR by determining 3′ splice site usage, showing that both core and auxiliary factors contribute to splicing outcomes [21]. IR is also modulated in response to environmental signals, such as cold stress in plants, via splicing factor responses mediated by chloroplast-dependent pathways [20,21,23,25,38,39,40].

### 2.2. Epigenetic Regulation

Chromatin structure is a key regulator of IR, with chromatin state models outperforming DNA-only models in predicting IR events [41]. IR is strongly associated with specific chromatin features such as DNase I hypersensitive sites and extended H3K4me3 histone marks, especially in short first introns [41,42]. These marks may reflect clustered nucleosomes and support a functional connection between IR, long non-coding RNAs, and higher-order chromatin organization [35,42,43]. Additionally, cell-specific epigenetic landscapes, including chromatin accessibility and histone modifications, regulate IR independently of gene expression levels [3,43,44]. The role of chromatin structure in shaping IR patterns is particularly relevant during development and cell differentiation.

**Figure 3:**
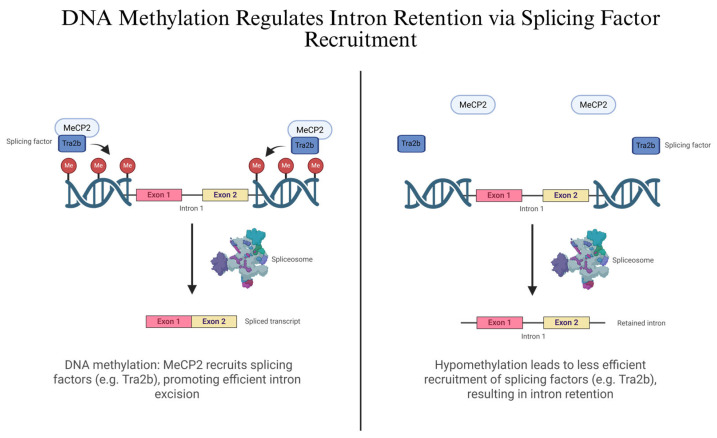
DNA Methylation Regulates Intron Retention via Splicing Factor Recruitment. This figure illustrates how DNA methylation influences IR by regulating the recruitment of splicing factors. (**Left panel**): DNA methylation allows MeCP2 to bind and recruit the splicing factor Tra2b, facilitating spliceosome assembly and efficient intron excision, resulting in a fully spliced mRNA transcript. (**Right Panel**): Hypomethylation leads to less efficient recruitment of splicing factors, resulting in IR. Created in BioRender. Ana L. Porras-Tobias. (2025) https://app.biorender.com/illustrations/687a5426628f4dc88167e093.

DNA methylation plays a critical role in regulating IR, particularly through its interaction with splicing factors [39]. Hypomethylation of intronic regions correlates with increased IR, while methylation recruits MeCP2, which facilitates the binding of splicing factors such as Tra2b, reducing IR (Figure 3) [26,45]. The S-adenosylmethionine (SAM) cycle also influences IR, with SAM levels modulating methylation and thereby impacting splicing of genes like *MAT2A* [46]. In rice, depletion of the histone methyltransferase SDG725 alters IR in a position-specific manner, further linking chromatin methylation to splicing regulation [44]. Overall, these studies highlight a coordinated regulatory network in which DNA and histone methylation, methyl donors, and splicing factors dynamically shape IR patterns across species and conditions.

### 2.3. Kinetic Coupling

**Figure 4:**
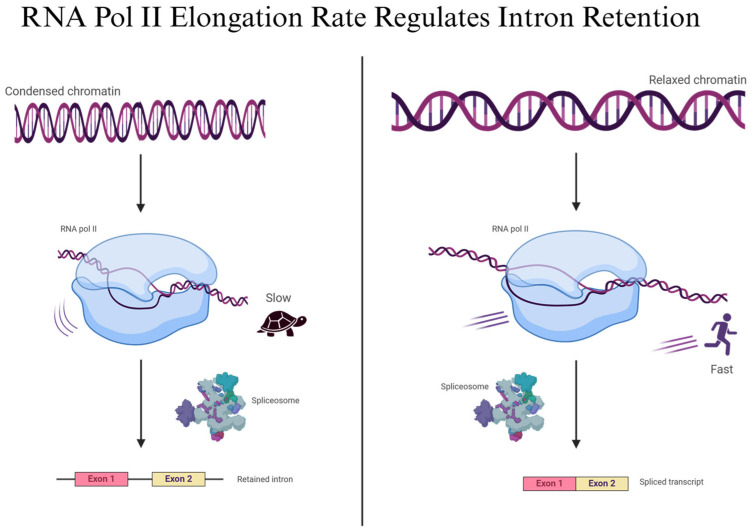
RNA Polymerase II Elongation Rate Regulates Intron Retention. This figure illustrates how the transcriptional elongation rate influences IR. (**Left panel**): Slow RNA polymerase II elongation, often due to condensed chromatin, delays spliceosome recruitment and increases IR, particularly in long genes. (**Right Panel**): Normal elongation allows timely spliceosome assembly and efficient intron removal, promoting canonical splicing. Created in BioRender. Ana L. Porras-Tobias. (2025) https://app.biorender.com/illustrations/687a6cf24bf3c8bf2de4742d.

Studies show that the RNA polymerase II elongation rate influences IR by affecting the timing of spliceosome recruitment and splice site recognition, especially in long genes [3,6,40,45,47,48,49,50,51]. Transcriptional splicing is shown to occur co-transcriptionally, with chromatin structure and polymerase pausing contributing to IR regulation, particularly under stress or during neuronal activation [49]. The spliceosome’s access to pre-mRNA is also regulated by nuclear condensates, as seen with CLK2 kinase, which can reorganize nuclear speckles and increase IR in cancer cells [47,52]. Reduced availability of spliceosomal components or delayed recruitment, coupled with RNA polymerase II stalling, acts as a checkpoint that promotes IR (Figure 4). In the instance of the spliceosomal component ZRSR2, the lack of the correct pre-mRNA transcript interferes with the spliceosome recognizing the 3’ splice site, which encourages IR [53]. Altogether, the study emphasizes that IR is tightly coupled to the transcriptional machinery, with dynamic interplay between elongation kinetics, spliceosome activity, and environmental or developmental signals shaping the IR landscape.

## 3. IR Functions as a Regulator of Gene Expression

In various organisms, from plants to fungi to mammals, IR accounts for transcriptomic complexity, controls gene output, and increases proteomic diversity.

Rather than being splicing errors and requiring repair, retained introns frequently serve a purpose. They can direct the fate of transcripts with variable nuclear retention, cytoplasmic decay via NMD, or delayed activation through post-transcriptional splicing. The dynamic control of mRNA availability for repression, storage, or the synthesis of novel protein isoforms with particular functions is made possible by these regulatory roles [14].

IR is regulated by sequence features including short intron length, weak splice sites, and GC content, and also influenced by epigenetic mechanisms including chromatin structure, RNA polymerase II elongation rate, and DNA methylation [39,45].

### 3.1. Mechanisms of IR as a Regulator of Gene Expression

Now being recognized as a distinct regulatory mechanism, IR controls gene expression at numerous levels of RNA processing. Across diverse cell types, organisms, and physiological states, IR dictates whether and when a transcript will be translated into a functional protein.

One major regulatory role of IR is trapping transcripts in the nucleus. By retaining specific introns that are either stored or targeted for degradation, cells can retain partially spliced mRNAs within the nucleus, thereby preventing premature or inaccurate translation [14,54]. This buffering system allows transcripts to accumulate in a dormant, intron-retained state, ready to be rapidly processed and translated in response to external cues. For example, in quiescent muscle stem cells, IR is widespread and contains transcripts of activation pathway genes, which forms a poised reservoir for rapid gene induction upon injury or stimulation [55]. Therefore, IR forms an adaptive and dynamic layer of post-transcriptional regulation by which cells have the ability to perform gene expression fine-tuning in real time. In some cases, intron-containing transcripts can be stored in the cytoplasm, which may act as “sentinel RNAs” capable of producing alternative RNA variants in response to stimuli [56].

IR also contributes to gene repression in the cytoplasm. Transcripts with retained introns tend to introduce PTCs, marking them for NMD [14,15]. This targeted degradation ensures elimination of incomplete or improperly processed RNAs and serves as protection against the non-functional translation of proteins [15].

Beyond repression, IR can redirect transcript function by modifying the 3’ UTR. These alterations affect polyadenylation, mRNA stability, and microRNA binding, ultimately impacting translational efficiency and protein output [17]. In certain cases, retained introns have also been shown to create novel protein isoforms with distinct or even opposing functions to canonical proteins. For example, hypoxia-induced retention of intron 12 in the *EIF2B5* gene leads to a truncated isoform that suppresses global translation and enhances tumor cell survival [57]. This illustrates the manner in which IR is not merely reducing gene expression, but it can actively reprogram protein function.

**Figure 5:**
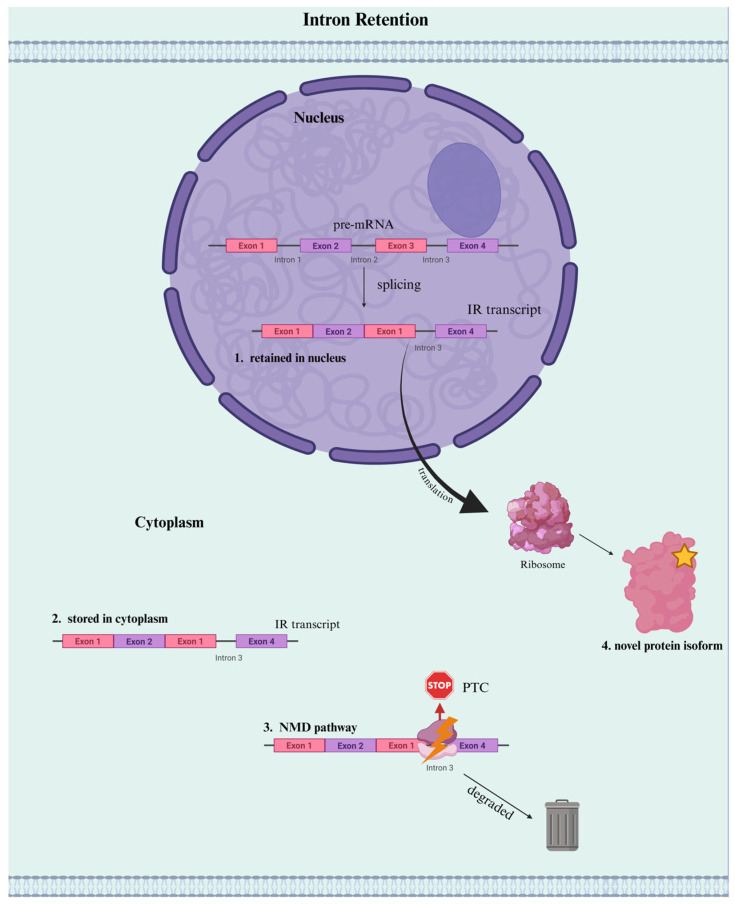
Outcomes of Intron Retention in Gene Regulation. When an intron is retained during mRNA processing, it can significantly alter transcript fate. The intron-containing mRNA may undergo nuclear retention (shown as #1), preventing export and translation, or be stored in the cytoplasm (shown as #2) in a translationally inactive form. Alternatively, retained introns often introduce premature termination codons (PTCs) that trigger nonsense-mediated decay (NMD) (shown as #3), reducing transcript abundance, or lastly, they can be translated into truncated or novel protein isoforms (shown as #4). Created in BioRender. Ana L. Porras-Tobias. (2025) https://app.biorender.com/illustrations/6875ed545c9667b402fb7ba0?slideId=7f2d599d-babb-4b78-a3ba-86dde5857487.

Taken together, these findings establish IR as a dynamic mechanism of gene regulation, in contrast to canonical mRNA processing where all the introns are removed accurately (Figure 1). Whether silencing transcripts, delaying their activation, or generating a novel isoform, IR adds a flexible layer of post-transcriptional control to the gene expression landscape (Figure 5).

### 3.2. Dynamic Role of IR in Cell Differentiation

IR is a dynamic regulatory mechanism that plays essential roles during cell differentiation across multiple organisms and developmental systems [20,42,58,59]. During granulopoiesis, IR in genes like *LMNB1* contributes to cell fate decisions via NMD, while in neuronal differentiation, downregulation of PTBP1 permits splicing of previously retained introns, enabling gene expression necessary for maturation [30,60,61]. IR is also involved in temperature-dependent sex determination, where its modulation in genes like *Kdm6b* and *JARID2* influences sex-specific developmental outcomes, and chromosomal sex determining regulation in Drosophila [62,63,64]. In T lymphocyte development, the RNA-binding protein hnRNPLL directs stage-specific IR patterns in immune signaling genes (Table 1) [28]. Altogether, IR operates as a developmentally responsive mechanism, finely tuning gene expression and protein output through the regulated retention or removal of specific introns [65].

### 3.3. IR Role in Stress and Stimulus Response

Interestingly, IR is a regulated response to environmental and physiological stress across species, rather than a splicing error [44,66]. Under heat shock and other stressors, proteins like CLK2 form nuclear condensates that hinder spliceosome function and promote IR in stress-response and tumor suppressor genes [47,52]. In plants and animals alike, stress-induced IR modulates gene expression by retaining transcripts for nuclear degradation, preventing translation, or producing non-functional or alternative protein isoforms [31,48,67]. IR dynamics are also influenced by epigenetic changes such as H3K36me3 enrichment, which correlates with suppressed translation under stress. These findings position IR as a critical post-transcriptional mechanism for regulating gene function during environmental challenges and cellular adaptation [9,40,59].

### 3.4. Role of IR in Cell-Type-Specific Gene Expression Programs

IR is tissue- and cell-type-specific, serving as a mechanism for regulating gene expression programs [6,32]. IR patterns are influenced by epigenetic marks and chromatin structure, which vary across cell types and operate independently of overall gene expression levels [43]. In tissues like liver, kidney, and neurons, changes in splicing factors like CTCF haploinsufficiency or PTBP1 reduction alter IR selectively in genes tied to functions such as metabolism, cytoskeletal organization, or neurodevelopment [30,31,68]. Cell-type-specific IR also regulates the expression of genes, such as *apoE*, in neurons, where intron-3 retention suppresses translation, revealing a potential therapeutic target for diseases like Alzheimer’s [69]. Overall, IR contributes to the precision and plasticity of tissue-specific gene regulation by enabling differential transcript usage tailored to cellular identity and function.

#### 3.4.1. Neurons

IR in neurons acts as a dynamic regulatory mechanism that enables rapid post-transcriptional responses to neuronal activity [32]. IR allows transcripts to remain in a stable, unspliced state until triggered by stimulation, such as NMDA receptor signaling, which initiates splicing, export, and translation [30,49]. Neuron-specific IR events, such as in apoE and BKCa transcripts, regulate protein expression critical for synaptic plasticity, stress response, and neuroprotection [69]. Furthermore, IR dysregulation is implicated in neurodegenerative diseases like ALS and spinal muscular atrophy, where defects in splicing factors or retention patterns disrupt gene expression and contribute to motor neuron pathology (Table 1) [33,58,70]. These findings position IR as a cell-type-specific, signal-responsive regulator of neuronal gene expression with both physiological and pathological relevance.

#### 3.4.2. Immune System

The regulatory role of IR in the immune system includes gene expression, RNA stability, and immune responsiveness [71,72]. LUC7L2 promotes intron 3 retention in the *MITA* gene, acting as a negative regulator of innate immunity by dampening type I interferon responses, particularly after viral infection [73]. Similarly, BUD13 controls IR in small, GC-rich introns, and its deficiency increases IR in immune genes like *Irf7*, impairing antiviral defense [18]. The splicing factor Lark/RBM4 also modulates IR during gut immune responses, affecting survival outcomes in infection [74]. These findings position IR as a feedback mechanism of immune activity, with implications for infection response, inflammation, and immune-related disorders.

#### 3.4.3. Heart

IR plays a functional role in cardiac gene expression and stress responses. In the heart, the *Hltf* gene undergoes IR to generate a truncated isoform that acts as a transcription factor, influencing Hif-1α–mediated transport systems and linking IR to epigenetic regulation through DNA methylation status [39]. The *ankrd1* gene also exhibits IR-producing isoforms that are co-expressed with intronless variants in a chamber-specific and stress-responsive manner in cardiomyocytes [75]. In a model of diastolic heart failure, ANKRD1 spliced and intron-retaining transcripts are upregulated, suggesting a self-regulatory mechanism that adjusts isoform balance under cardiac stress [75]. These findings underscore IR as a key contributor to functional transcript diversity in the heart, which is relevant to both development and disease [39,75].

## 4. Detection and Quantification of IR

RNA sequencing (RNA-seq) is a powerful tool for studying IR. However, there are several series of biological and technical challenges that limit precise quantification and analysis. IR events occur at low frequencies and show high variability across tissues, suggesting tight regulation and cell-type-specific splicing programs [76]. The intronic regions involved are often long, GC-rich, and filled with repetitive sequences such as transposable elements (e.g., LINEs and SINEs), making read mapping difficult due to multi-mapping, which introduces “noise” and biases [4,76,77]. These technical obstacles are further exacerbated by misaligned reads, antisense transcription, and potential DNA contamination [78].

For a comprehensive discussion of these limitations, Vanichkina et al. (2018) offer an especially detailed and insightful breakdown of both the biological and computational challenges involved in IR detection and quantification.

### 4.1. Sequencing Technologies: Short- vs. Long-Read Approaches

Now, the choice of sequencing technology greatly shapes how IR is detected, quantified, and interpreted across diverse biological contexts. Short-read and long-read sequencing platforms offer distinct approaches to IR analysis.

#### 4.1.1. Short-Read Sequencing

Between short-read and long-read sequencing, short-read is the most popular sequencing technology. Short-read sequencing (e.g., Illumina) is a type of Next-Generation Sequencing that involves the fragmentation of DNA or RNA into relatively shorter sequences and is well-supported for transcriptomics while being affordable. The only limitation is that this technology struggles to determine how IR events are combined into isoforms due to the fragmentation of RNA that occurs prior to sequencing [79].

#### 4.1.2. Long-Read Sequencing

Whereas long-read sequencing (e.g., PacBio and Nanopore) has the advantage that it can capture full-length transcripts and minimize the need for inference. The Nanopore technology method applies to single-cell transcriptomes as it provides the quantification of mRNA and AS information at the single-cell level. Nanopore long-read sequencing, combined with the target capture of full-length double-stranded cDNA sequencing, has been successfully used to detect IR events. It can also accurately identify splicing variants and mutations in clinical samples, such as novel *TSC2* gene intron mutations producing truncated proteins [79]. However, long-read sequencing is limited by sequencing depth and accuracy [4].

### 4.2. Computational Tools

IR quantification from RNA-seq data requires specialized tools that address the unique features of retained introns. The following table highlights some widely used and well-established computational tools for detecting and analyzing IR, each suited to different experimental and sequencing contexts (Table 2).

Building on this comparative overview, it is important to notice a shared constraint across nearly all IR detection tools: the inherent biases introduced by intronic complexity and sequencing limitations. As mentioned previously, introns are significantly longer than exons and more prone to overlapping genomic features. Consequently, most computational approaches introduce a selection bias, as only introns with sufficient coverage can be detected. This skews IR quantification, disproportionately favoring the more expressed genes [78]. As sequencing technologies continue to evolve, addressing these biases remains vital for refining our understanding of IR in both health and disease.

### 4.3. Quantitative Metrics for IR

Two primary metrics are employed for IR quantification.

#### 4.3.1. IR Ratio

The IR Ratio is used to quantify the degree of IR by measuring how frequently introns are retained within mature mRNA transcripts instead of being correctly spliced out.

$$\mathrm{IR}\;\mathrm{Ratio}=\frac{Intronic\;Abundance}{Intronic\;Abundance+Normal\;Splicing\;Abundance}$$

where Intronic abundance is measured using the average or median read depth across the intron. The abundance of normal splicing is assessed by counting the number of reads that span the exon-exon junctions flanking the intron [78].

#### 4.3.2. Percent Spliced-In (PSI)

The second metric is PSI, where it measures the proportion of mRNA transcripts in which a specific intron is retained, as opposed to being spliced out during RNA processing.

$$\mathrm{PSI}=\frac{IntronTPM}{IntronTPM+non-IR\;transcript\;TPMs}$$

#### 4.3.3. PSI vs. IR Ratio

Quantifying splicing efficiency is difficult since exons flanking a given intron can participate in multiple isoform configurations, connecting not only to each other, but also to other exons within the same gene. Because of this, it makes it complicated to determine how many reads truly support intron excision. The metrics described above (IR Ratio and PSI) approach this issue differently.

IR Ratio estimates IR by looking only at reads that span the junctions of the exons on either side of the intron. It takes the higher number of reads from either side to reduce confusion caused by different transcript isoforms. This helps avoid bias from changes in the overall gene expression, but results may be unreliable when read coverage is low [78].

On the other hand, the PSI method uses read coverage from the whole transcript, which leads to higher stability when read counts vary across different regions. However, PSI may be less accurate for genes that contain many AS events, as it is harder to know which transcripts the reads originated from. Each method has its strengths and weaknesses depending on the gene’s structure and the quality of the data [78].

## 5. Diseases Associated with IR

IR has emerged as a key contributor to disease pathogenesis by a variety of distinct mechanisms. Retained introns can introduce PTCs, destabilizing the transcript by NMD. Alternatively, they can allow translation of aberrant isoforms that are truncated, misfolded, or toxic to the cell. In some cases, IR events are the results of mutations that interfere with normal splice sites or activate cryptic ones, while in others they are secondary to epigenetic dysregulation or environmental triggers such as stress, hypoxia, or inflammation. Together, these mechanisms position IR as a central regulator of gene expression, capable of altering cellular function and driving disease progression.

Given its diverse molecular consequences, IR contributes to a wide range of pathological conditions. In this section, we examine several biological systems where IR plays a mechanistic or modulatory role. These include aging, neurodegenerative disorders, oncogenic processes and tumor immunogenicity, the breast cancer paradox, developmental and sex differentiation disorders, and fibrotic disorders (extended in Table 3).

### 5.1. Aging

Aging is accompanied by widespread molecular changes, including alterations in RNA splicing. IR affects genes involved in metabolism, stress response, and longevity regulation, and may serve as an early biomarker of aging.

- Spliceosomal Decline and IR Accumulation: Aging alters the expression and localization of core splicing factors such as SF3B1, SRSF proteins, PUF60, and ASF/SF2, compromising spliceosome fidelity. This leads to increased IR in genes like *POLR2A*, impairing global transcription and promoting cellular senescence. XAB2 depletion further induces IR in *POLR2A*, linking splicing failure to aging processes [7,16,101,102].
- Sensor Gene IR in Pre-Symptomatic Aging: In klotho and SAMP8 mouse models, IR accumulates in stress-responsive genes such as *Nr1h2* and *Slc16a3* (*MCT4*) during early aging stages, particularly in the hippocampus. These events precede neurodegeneration and may serve as early biomarkers of age-related brain dysfunction [86,87].
- Transcriptional Readthrough and IR Coupling: Aging and senescence are associated with increased transcriptional readthrough and IR, specifically in the mouse hippocampus and human prefrontal cortex. These defects are linked to altered RNA polymerase II dynamics and elevated transposon expression, indicating transcriptional dysfunction [38,88].
- Therapeutic Reversal by Kampo Medicine: The traditional Japanese herbal medicine Juzentaihoto (JTT) restores normal splicing patterns in aging models by reversing IR in sensor genes. This intervention improves metabolic regulation and may serve as a functional marker for anti-aging therapies [16,87].

### 5.2. Neurodegenerative Disorders

#### Alzheimer’s Disease (AD)

IR has been recognized as a critical molecular mechanism involved in the pathogenesis of AD. The following studies highlight multiple distinct IR events that control transcript and protein levels in the brain, which affect disease progression:

- *DDIT4L* IR (DIR): Retention of an intron in the *DDIT4L* gene produces a toxic isoform known as DIR. This protein promotes amyloid β aggregation when interacted with gelsolin, accelerating plaque formation. When bonded with GluA1, an AMPA receptor subunit, it leads to synaptic dysfunction, which contributes to cognitive decline. Hypoxia was shown to enhance DIR expression, suggesting that environmental stressors can trigger or worsen IR-mediated AD pathology [98].
- Sex-Biased IR Patterns: Females with AD exhibit 1645 elevated IR events compared to 80 in males, particularly affecting genes involved in ubiquitin signaling and Tau protein binding, which are critical in AD pathology. These events are associated with lower mRNA levels via NMD and are regulated by epigenetic markers such as H3K27ac and CTCF near splice sites, implicating at the chromatin level control of IR [103].
- IR in gene *Slc16a3* (MCT4): In the AD mouse model and aged brains, there was found a retention of intron 2 between exons 2 and 3 in the *Slc16a3* gene in astrocytes and endothelial cells, especially under inflammatory stimulation. This variant may indicate disruption of both the lactate transport and metabolic regulation [86].
- Tau11i from IR in *MAPT*: Retention of intron 11 in the *MAPT* gene is associated with the synthesis of a truncated protein isoform, Tau11i, that accumulates in AD brain regions. Tau11i displays features shared with full-length Tau441: altered aggregation, higher stability, and reduced microtubule binding. However, Tau11i weakly co-localizes with α-tubulin and Tau fibrils, which drives early neurodegenerative progression [99].

### 5.3. Oncogenic Mechanisms and Tumor Immunogenicity

#### 5.3.1. Ovarian Cancer (OC)

OC encompasses several different subtypes, and one rare type: Small Cell Carcinoma of the Ovary, Hypercalcemic Type (SCCOHT), which stands out for its unique pattern of RNA splicing. In many forms of OC, IR contributes to tumor development by changing gene expression patterns, creating abnormal proteins (neoantigens), and disrupting the activity of splicing-related proteins.

- *SMARCA4* Loss in SCCOHT: In SCCOHT, the loss of gene *SMARCA4* is seen to induce widespread IR and exhibit tumor-specific splicing patterns. Mass spectrometry confirmed IR-derived peptides capable of MHC-I binding and T cell activation, suggesting that IR could be a source of neoantigens and a promising target for immunotherapy [89].
- *WBP11* and *MCM7* IR Suppression: In OC, overexpression of the splicing factor WBP11 prevents the retention of intron 4 in the *MCM7* gene, leading to increased cancer cell proliferation. When *WBP11* is silenced, IR is restored, *MCM7* expression is reduced, and tumor growth is suppressed, making WBP11 a potential therapeutic target [104]. This mechanism reveals how splicing factor dysregulation can drive oncogenesis by repressing IR.
- *CD44* Intron 9 Retention as a Biomarker: Abnormal retention of intron 9 in *CD44* mRNA is seen in 60% of OC cell lines, but not in normal ovarian tissue. This intron retention may disrupt CD44 isoform expression and contribute to tumor development, making it a potential diagnostic marker for OC [105].

#### 5.3.2. Breast Cancer Paradox

Breast cancer presents a unique IR pattern compared to other malignancies, characterized by lower IR levels in tumors than in adjacent normal tissue, in contrast to the elevated IR commonly seen in most other solid or hematological cancers.

- High Baseline IR in Normal Breast Tissue: Pan-cancer analyses have shown that normal breast tissue exhibits the highest IR levels among all tissue types. This elevated baseline explains why breast tumors show reduced IR, despite IR being generally increased in other cancers [90].
- Prognostic Implications in the Luminal B Subtype: Within breast cancer subtypes, the luminal B subtype shows a link between IR levels and poor prognosis. Lower IR is associated with higher cell proliferation, suggesting that rapidly dividing tumor cells may suppress IR to streamline gene expression and support faster growth [90].
- Epigenetic Influence and Population Differences: In breast cancer, DNA hypomethylation of introns is linked to increased IR. Comparative analysis shows that African-American patients have lower intronic methylation and higher levels of retained intron expression compared to European-American patients, suggesting that the epigenetic regulation of IR may vary across populations [26].

### 5.4. Developmental and Sex Differentiation Disorders

#### 46,XX DSD

IR contributes to sex differentiation disorders by altering protein isoform balance and affecting gene regulation during gonadal development. 46,XX DSD is a rare condition in which individuals with a common female chromosome pattern (46,XX) develop male external genitalia [94].

- *WT1* Intron 9 Retention in 46,XX DSD: A novel splice-site mutation (c.1437A > G) in the *WT1* gene causes retention of intron 9, which produces a truncated +KTS isoform lacking zinc finger 4 (ZnF4) and fails to express the production of the -KTS isoform. This imbalance interferes with *WT1*’s role in controlling gene activity and disrupts normal sex determination, resulting in testicular or ovotesticular development in a genetically 46,XX individual [94].

### 5.5. Fibrotic Disorders

#### Renal Fibrosis

Fibrotic disorders are characterized by excessive extracellular matrix deposition, often influenced by dysregulated signaling pathways and transcriptomic alterations such as IR.

- Renal Fibrosis and TGF-β co-receptor endoglin (ENG) IR Modulation: In chronic kidney disease, ENG exists in two isoforms. One promotes fibrosis and a shorter one that protects against it. This study used antisense oligonucleotides to trigger terminal intron retention in ENG pre-mRNA, shifting splicing toward the anti-fibrotic short isoform. Patient biopsies showed that overall ENG levels were high, but the protective short isoform was reduced. Antisense oligonucleotide treatment restored short-ENG expression and reduced TGF-β1-driven pro-fibrotic genes and proteins such as ACTA2, COL1A1, and FN1. These results suggest that modulating IR can be a promising strategy to treat renal fibrosis by enhancing protective splicing outcomes [95].

## 6. Discussion

Although there have been many breakthroughs in research concerning IR, the intricate nature of IR and its functional consequences remain challenging to fully characterize. There are functional roles of IR in both enhancing and repressing gene expression across cellular contexts. IR can enhance transcriptomic and proteomic diversity through cytoplasmic intron-containing transcripts, which may serve as “sentinel RNAs” capable of generating alternate RNA variants in response to stimuli [56]. IR can also repress gene expression by preventing mRNA export and triggering nuclear degradation via RNA surveillance pathways, as shown with PTBP1, which retains terminal introns in non-neuronal cells [30]. Upon neuronal differentiation, PTBP1 is downregulated, allowing these introns to be spliced out and enabling translation, illustrating IR’s role in precise developmental gene regulation [30]. Overall, IR functions as a context-dependent regulatory switch, balancing mRNA stability, localization, and translational readiness [30,56].

In addition, IR is a versatile gene regulatory mechanism that often leverages NMD to maintain transcriptome integrity and cellular function. NMD is a critical pathway through which IR regulates gene expression [6]. IR frequently introduces PTCs that trigger NMD, reducing the levels of aberrant or unnecessary transcripts in diverse contexts like granulopoiesis, stress responses, and tumor suppression [11,63,106,107]. In plants and mammalian systems, IR-NMD mechanisms fine-tune protein output, with specific examples including LMNB1, Chtop, and OsGolS, where retention leads to transcript degradation [34,48,60]. However, not all intron-retained transcripts undergo NMD; some remain stable or are regulated by alternative pathways such as nuclear sequestration or microRNA targeting [30,35,108,109].

Another key regulatory pathway of IR is nuclear retention, which prevents premature or inappropriate mRNA translation and establishes IR-linked nuclear retention as a versatile mechanism for controlling transcript availability, localization, and function across diverse biological systems [11,30,35]. IR transcripts often contain PTCs or altered 3′ UTRs that inhibit nuclear export, as seen with METTL3 and apoE variants [69,110]. Proteins like PABPN1 help retain these intron-containing RNAs in the nucleus by coupling 3′ end processing with splicing regulation, while stress conditions like heat shock induce widespread IR and transcript sequestration [49,111,112]. Nuclear retention serves as a post-transcriptional checkpoint, modulating gene expression dynamically, particularly during cellular stress or differentiation [6,47,60,113].

The production of biologically active isoforms through IR emphasizes the functional consequences of spliceosome-mediated decisions. These findings present how RBPs regulate gene expression by combining IR with cell-specific functions, stress responses, and disease processes [3,26,27,28,30,31,32,33,34,35]. Furthermore, IR is key to generating the diverse mRNA isoforms, many of which encode truncated proteins with altered regulatory functions [11,39,114,115,116,117]. IR can introduce PTCs, as seen in genes like *METTL3*, *EIF2B5*, and *Notch4*, producing truncated isoforms that suppress translation or modify cellular responses, such as stress adaptation or immune modulation [31,48,57,110,118]. In certain cases, such as ANKRD1 in the heart or endoglin in endothelial cells, both full-length and intron-retained isoforms are co-expressed, enabling context-dependent gene regulation [7,75].

IR also influences disease-related genes, where retained introns can inactivate tumor suppressors or generate oncogenic protein variants [52,106,119,120,121]. IR contributes to tumor progression by generating truncated oncogenic proteins, suppressing tumor suppressor genes like *TIMP1* and *DMD*, and enabling cancer cells to adapt to stressors like hypoxia [47,121,122,123]. Epigenetic factors, such as H3K36me3 enrichment, and external stimuli like heat shock, further modulate IR, often leading to altered splicing patterns that support cancer cell survival [47,52,57,113]. IR also generates neoantigens, with potential as biomarkers for prognosis in cancers like multiple myeloma and pancreatic cancer [72,124]. Collectively, these findings highlight IR as a key mechanism for increasing proteomic diversity and actively regulating gene function through the production of both functional and non-functional isoforms, serving as an essential regulatory mechanism of human pathology. This is evident in the case of cancer biology, where it affects gene expression, immune evasion, and tumor adaptability [26,43,47,106,119,125,126].

To improve our understanding of IR, future research should focus on addressing several key obstacles. First, advancing computational tools and RNA sequencing technologies to better handle the repetitive genomic sequences in introns, which can confound accurate mapping, especially with long reads [4]. Second, improved sequencing protocols that integrate both junction reads and those mapping to intronic regions are needed to overcome limitations in coverage and mappability, especially in regions with high repetitive content [4]. Third, the phenomenon of NMD presents an obstacle to detecting IRTs, as many of these transcripts are rapidly degraded in real time [4]. Finally, these challenges will offer a new era of AS research, opening up a deeper understanding of gene expression regulation at another level [4].

## 7. Future Directions

New methods for integrating long-read sequencing data with short-read data must be developed. Long-read technologies, although promising, face scalability challenges when applied to complex genomes or large datasets. Repetitive elements such as LINEs, SINEs, and DNA transposons, which are more abundant in introns than exons, complicate read mapping, leading to inaccurate estimates of IR levels [4]. Standard protocols for IR detection, quantification, and normalization, akin to those available for differential gene expression analysis, will be critical for standardizing IR research.

Studies that examine the relationship between IR and NMD mechanisms will help address the underestimation of IRTs in RNA-seq analyses. There need to be computational and experimental tools for investigating the regulatory mechanisms of IR, its impact on protein isoform generation, RNA-based regulation, and potential degradation pathways to uncover the functional roles of IR.

## Figures and Tables

**Table 1:** RNA Binding Protein functions, sequence patterns, and highlights. This table demonstrates only a few key RBPs found during the IR research overview. The second column presents the function of these RBPs with the process of IR. The third column states the sequence pattern found that accompanies IR with its specific RBP. The fourth column includes normal regulatory gene expression through IR and the key role that IR has in the pathogenic process. These RBPs reveal a pattern but do not answer the question of why some introns are retained and others are not.

RNA Binding Protein	Key Function	Binding to Specific RNA Sequences	IR Highlights	References
Polypyrimidine Tract-Binding Protein 1 (PTBP1)	Regulator of alternative splicing	Pyrimidine-rich RNA sequences, particularly those rich in cytosine (C) and uracil (U) bases near splice junctions	Represses splicing of terminal introns to retain transcripts in the nucleus	[31]
Splicing Factor Proline and Glutamine Rich (SFPQ)	Splicing factor, particularly important for splicing long genes, and regulates the formation of circular RNAs (circRNAs)	RNA sequences, particularly those surrounding cryptic last exons and in long introns.	SFPQ exports IR transcripts to the cytoplasm that is associated with ALS	[33]
Heterogeneous Nuclear Ribonucleoprotein L Like (hnRNPLL)	Regulates alternative splicing	5′-YCAY-3′	Regulates lineage-specific IR during T cell development	[28]
Chromatin Target of PRMT1 (Chtop)	Component of the TREX (TRanscription-EXport) complex, which links transcription to mRNA export	Interacts with RNA through its arginine-glycine-rich (RG) domain and its N-terminal (N1) domain	Regulates Chtop mRNA expression antagonistically under specific stimuli	[34]
Poly(A) Binding Protein Nuclear 1 (PABPN1)	mRNA processing and export	Poly(A) tails of mRNA molecules	*PABPN1* gene mutation results in oculopharyngeal muscular dystrophy	[27]
NOVA Alternative Splicing Regulator 1 (Nova-1)	Regulates alternative splicing	5′-YCAY-3′	Regulators in certain mammalian neurons	[33]
ASF/SF2 (SRSF1)	Constitutive and alternative pre-mRNA splicing	Exonic splicing enhancers and 5’ splice sites	Specifically involved in the synthesis of endoglin	[7]

**Table 2:** Summary of computational tools for intron retention detection and analysis. Tools vary in their methodology and sensitivity. Selection should be guided by experimental design, data type, and analysis goals.

Tool/Approach	Highlights	References
IRFinder/IRFinder-S	Benchmark IR detection tool; IRFinder-S adds CNN filtering, long-read support, and integrated differential IR analysis	[78,80,81]
iREAD	Uses entropy scoring across independent introns to detect flat read distributions; avoids exon overlap	[82]
KMA	Involves transcript quantification; suitable for differential analysis with minimal artifacts	[78,82]
IntEREst	Supports non-annotated introns; integrates statistical tests for intra- and inter- sample comparisons	[53,78]
IRAVNet	Identifies IR-causing variants directly from transcriptomic data; assesses the connection between genomic variant status and the amount of splicing changes	[83]
SpliceAI/SpliceAI-10k	Deep learning model for splicing prediction; identifies partial IR and spliceogenic variants with high sensitivity	[84,85]

**Table 3:** Comparative Overview of IR-Linked Disease Mechanisms. Highlights distinct IR features, pathways, and gene targets across major disease categories.

Disease	Key IR Feature	Gene(s) Involved	Functional Consequences	References
Aging	Increased IR in genes	Various	Impaired metabolic homeostasis; reversible by JTT, which may serve as a functional marker for anti-aging therapies	[16,38,86,87,88]
SCCOHT (Ovarian Cancer)	SMARCA4 loss triggers IR → neoantigens	*SMARCA4*, MHC-I pathway	IR-derived peptides activate T cells; opens immunotherapy avenues in chromatin-remodeling cancers	[89]
Breast Cancer	IR globally decreased; low-IR tumors linked to poor prognosis	Various	Contrasts most cancers; IR levels correlate with proliferation and prognosis	[90]
Hepatocellular Carcinoma	IR generates a non-coding isoform (KLF-003) that is downregulated	KLF4	Epigenetic silencing of IR transcript via CpG methylation; loss may contribute to recurrence and could serve as a diagnostic biomarker	[91]
Multiple Myeloma	Elevated IR events → generation of IR-neoantigens	RNA splicing machinery; immune checkpoint pathways	High IR-neoAg load correlates with poor survival, immune suppression via co-inhibitory molecules, reduced MHC-II, and immune escape	[92]
Etoposide-Resistant Leukemia	Retention of intron 19 in TOP2α mRNA	*TOP2α* gene (DNA topoisomerase Iiα)	Production of truncated TOP2α/90 isoform, reduced drug induced DNA damage; chemoresistance	[93]
46,XX DSD	IR in *WT1* alters isoform ratio → sex development disruption	WT1	First documented IR-induced mechanism affecting human sexual differentiation	[94]
Renal Fibrosis	ASO-induced IR in *ENG* shifts isoform balance to anti-fibrotic variant	ENG	Demonstrates therapeutic modulation of IR to treat fibrosis via isoform control	[95]
Incessant Ventricular Tachycardia	Retention of 79 bp from intron 14	*SCN5A* (Nav1.5 sodium channel)	PTC (p.R818 *) → truncated protein → impaired sodium channel function → arrhythmia	[96]
Allgrove Syndrome	Partial retention of 99 bp of intron 14 plus exon 14 skipping	AAAS	Frameshift mutations causing PTCs, likely resulting in non-functional protein and disease phenotype	[97]
Alzheimer’s Disease	IR in *DDIT4L* and *MAPT* produces toxic isoforms (DIR and Tau11i)	DDIT4L, MAPT	Links IR to neurodegeneration, synaptic dysfunction, and cognitive decline	[98,99]
Hypoplastic Amelogenesis Imperfecta	Retention of intron 1 and normally skipped exon 2 → elongated 5’ UTR	*ENAM* (enamelin)	Complex 5’ UTR secondary structure attenuates translation → reduced enamelin → defective enamel	[100]
ALS	Premature and increased IR during motor neuron differentiation; prominent IR in SFPQ transcript	*SFPQ*	Reduced nuclear SFPQ protein; disrupted RNA metabolism; ALS pathogenesis marker	[33]
SMA	Intron 7 retention in SMN2 transcripts	*SMN2*	Longer 3’ UTR → translational repression of SMN protein	[17]

(*) denotes a stop codon, which terminates translation, resulting in a truncated protein.
